# The feasibility of determining the impact of primary health care research projects using the Payback Framework

**DOI:** 10.1186/1478-4505-7-11

**Published:** 2009-05-08

**Authors:** Elizabeth C Kalucy, Eleanor Jackson-Bowers, Ellen McIntyre, Richard Reed

**Affiliations:** 1 Primary Health Care Research and Information Service, Department of General Practice, Flinders University, Adelaide, South Australia, Australia; 2 Discipline of General Practice, Flinders University, Adelaide, South Australia, Australia

## Abstract

**Background:**

Primary health care research is under pressure to be accountable to funders in terms of benefits for practice and policy. However, methods to assess the impact of primary health care research must be appropriate to use with the diverse topics, settings and approaches of this sector. This project explored the feasibility of using the Buxton and Hanney Payback Framework to determine the impact of a stratified random sample (n = 4) of competitively funded, primary health care research projects.

**Methods:**

The project conducted telephone interviews based on the Payback Framework with leaders of the research teams and nominated users of their research, used bibliometric methods for assessing impact through publication outputs and obtained documentary evidence of impact where possible. The purpose was to determine the effectiveness of the data collection methods and the applicability of the Payback Framework, and any other issues which arose around the assessment of impact of primary health care research.

**Results and discussion:**

The thirteen interviews were resource intensive to organise conduct and analyse but provided better information about impact than bibliometric analysis or documentary analysis. Bibliometric analysis of the papers published from the four projects was hampered by the inclusion of only one of the journals in major citation indexes. Document analysis provided more evidence of dissemination than of impact.

The payback framework and logic model were a sound basis for assessing impact. Chief investigators and nominated users of research provided substantial information relevant to the impact categories closest to their spheres of influence and awareness, but less about the impact their research had on the wider health sector, population health or economic benefits. An additional category of impact emerged from the interviews, that of strengthening research networks which could enhance the impact of later work. The framework provided rich information about the pathways to impact, better understanding of which may enhance impact.

**Conclusion:**

It is feasible to use the Buxton and Hanney Payback framework and logic model to determine the proximal impacts of primary health care research. Though resource intensive, telephone interviews of chief investigators and nominated users provided rich information.

## Background

Research funding in Australia has been under increased pressure to be accountable in terms of benefits to the wider community, with goals such as "Well informed primary health care practice and policy" [1] for the Australian Government's Primary Health Care Research Evaluation and Development (PHCRED) strategy. The Australian primary health care research sector has grown in strength and quality with the advent of the PHCRED strategy in 2000, but in a highly competitive funding environment its continued funding will be influenced by the perceived impact of research on practice and policy in this sector. This project was driven by the need to assess the impact of primary health care research, despite particular challenges due to the breadth of this complex sector with its diverse topics, settings, research methods and approaches.

A review of the literature on research impact [2] highlighted the multidimensional, unpredictable, non linear and contingent nature of research impact [3,4] and the risk that the considerable challenges associated with assessing gains in knowledge, wealth and health may lead to "counting what we can measure rather than measuring what counts" [5]. Several models were identified which could be used to assess research impact [2], the most promising being the Buxton and Hanney Payback Framework, which has been tested on several bodies of research, including Arthritis Research Council grants[6], National Health Service (NHS) funded research in the UK [7], and more recently on health and health services research in Hong Kong [8].

As the first phase of a larger program examining the impact of nationally funded Australian primary health care research, this project explored the use of the Buxton and Hanney Payback Framework [6]. The purpose was to determine the effectiveness of the data collection methods and the applicability of the Payback Framework, and any other issues which arose around the assessment of impact of primary health care research.

### Buxton and Hanney Payback Framework

The Buxton and Hanney Payback framework has five payback categories of benefits resulting from health research which can be used to structure case studies of the impact of research projects. The payback categories are shown with Buxton and Hanney's scope notes [6] in Table 1. Each category may be scored to provide a numeric comparison. The associated Logic Model (Table 2) depicts the interface between research team and the wider community at different stages in the research project.

**Table 1:** Availability of information for the assessment of research impact using Payback Framework categories

		**Project 1**	**Project 2**	**Project 3**	**Project 4**
Funding source		NHMRC	PHCRED	NHMRC	GPEP

Funding amount (Au$)		135,000	134,000	150,000	156,000

Completion Dates		2002–2004	2003–2005	1999–2000	2000–2002

Project methods		Action research	Case notes audit	RCT	Case note audit

Number of interviews		4	4	3	2

**Payback category**	**Scope**				

Knowledge production	Peer reviewed articles	✓	✓	✓	

	Listed in the ISI index			✓	

	Listed in Scopus	✓	✓		

	Citations	✓		✓	

	Journal impact factor			✓	
	
	Readership targeted by journal articles	✓	✓	✓	

Research targeting, capacity building and absorption	Better targeting of future research	✓	✓		✓

	Development of research skills, personnel and research capacity	✓	✓		✓

	Critical capacity to utilize appropriately existing research				✓

	Staff development and educational benefits	✓	✓	✓	

Informing policy and product development	Improved information base on which to take political and executive decisions	✓	✓		

	Clinical or regional guidelines		✓	✓	

	Education/training policies or audit and evaluation criteria,	✓	✓	✓	~

	Inclusion in systematic review				✓

	Inform product development	✓			✓

Health and health sector benefits	Cost reduction in the delivery of existing services				✓

	Qualitative improvements in process of service delivery	~	✓		

	Increased effectiveness of health services: increased health	✓	✓	✓	

	Equity: improved allocation of resources at an area level, better targeting, accessibility.		✓		~

	Revenues gained from intellectual property rights	✓			

Broader economic benefits	Wider economic benefits from commercial exploitation of benefits arising from R&D				

	Contribution to a healthy workforce	~	✓		

**Table 2:** Logic Model: Availability of information on research processes which potentially lead to impact

**Processes that lead to outcomes**	**Scope**	**Project**			
		**1**	**2**	**3**	**4**

Stage 0 Topic/issue identification	Generation of original idea	✓	✓		✓

Interface A Project specification and selection	Processes of development of proposal and submission	✓	X	X	X

Stage 1 Inputs to research	Other funding Experience of research team Knowledge base of team	✓	✓	✓	✓

Stage 2 Research processes	How appropriate research methods were Difficulties encountered How potential users were involved	✓	✓	✓	✓

Stage 3 Primary outputs from research	Types of publications Non conventional outlets for publications How follow on research happened How other take up of research happened Capacity building	✓	✓	✓	✓

Interface B Dissemination	Processes of uptake in policy/industry eg presentations, networking	✓	✓	✓	✓

Stage 4 Secondary outputs- policy making and product development-	Pathways to policy/product development	✓	✓		✓

Stage 5 Adoption by practitioners and public	Pathways to behavioural change by practitioners	✓			

Stage 6 Final outcomes	Pathways to health or economic benefits				

Data gathering methods used to assess impact in the Arthritis Research Council (ARC) study [6] included

• bibliometric analysis of the publications derived from the projects;

• analysis of documents associated with the project such as original proposals, reports, publications, conference presentations, newsletter articles, policy documents, media publications and more;

• semi structured telephone interviews with chief investigators and persons they nominated to provide further information about how the research was used in policy or practice.

In the ARC study, information about research projects gathered through these methods was compiled into a structured narrative, organised according to the Payback Framework. The Logic Model enabled comparison between projects, to make it possible to "look for common factors associated with research that has led to outcomes" and to "see how far such outcomes are associated with different modes of funding and types of research" [6].

## Methods

The feasibility of using the Payback framework to determine impact of primary health care research was explored by applying the data collection methods described in [6] with four projects. Four members of the research team then examined the resulting data to determine the extent to which the methods were useful to collect data about the impact of the research projects, the practicality of the methods in terms of time and effort, the applicability of the Payback Framework in terms of the extent to which the data fitted the impact categories, and any issues which arose around the assessment of impact of primary health care research. These data were entered on a grid and a consensus was arrived at by all members of the team after extensive discussion.

### Sample

The sample frame included all primary health care research projects funded competitively at national level by the National Health and Medical Research Council (NHMRC), General Practice Evaluation Program (GPEP) and PHCRED, to a minimum of A$100, 000, commenced in 1999 or later and completed by 2005 (n = 20). The definition of "primary health care" was that used by the chief investigators to describe their project.

These criteria were chosen to maximise the chance that the time frame would allow for the selected projects to have had an impact. At the same time, we wanted to ensure that respondents could be located and that projects were recent enough that respondents could fully recall their activities. We were guided by the finding by Butler and Biglia [9] that 99% of journal articles from projects are published over a period of up to seven years from the start of the project.

Stratified sampling was used. We randomly selected one randomised controlled study from the whole sample. One project was then selected randomly from each of the three funding bodies. This contrasts with the Arthritis Research Council impact study [6] where bibliometric analysis of publications was used to identify projects from the top and middle deciles in terms of publication numbers.

### Data collection

The data collection methods used were based on those used by Hanney et al in their study of Arthritis funding in the UK [6]. The methods were interwoven not sequential. Semi-structured interviews were conducted with chief investigators and nominated persons. Publications and documents relating to each project were collected, though original proposals and referees reports [6] were not available.

The interview schedule which was developed in consultation with the Advisory Committee incorporated categories of the Payback Framework [6] and questions about the dissemination strategies and interface with end users derived from the Logic Model. The questionnaire was adapted for end users of the project findings. (see appendix 1 for copy of interview schedule).

Chief investigators (CI) for each project were contacted by email, then telephone, seeking their informed consent to participate. The research officer and one other team member then conducted a semi-structured interview by telephone with each participant. Each chief investigator was invited to nominate other persons who could contribute to understanding how the project findings had been used. During interviews, chief investigators were asked to list publications derived from the project. The Institute for Scientific Information (ISI) Web of Science and Scopus [10,11] databases were used to locate these articles, ascertain the number of recorded citations, and identify the Impact Factor of the journals in which they were published.

Chief investigators were also asked about presentations, papers, media articles, reports, resources and other items which derived from their research project, and copies were obtained where possible. Internet searching using Google and Google Scholar was used to locate references to the projects.

Reliability was addressed by including multiple sources of data, types of data, and research projects, and thorough analysis of the data by multiple researchers. Clearance and validation were addressed by providing a draft copy of the research findings to participants for comment on how well the findings reflected their experience.

### Ethics

The Flinders University Social and Behavioural Ethics Committee granted ethics approval for the first stage of this project (Ref RSBRC 3616) in July 2006.

A national advisory committee was formed to advise the research team about project direction and oversee progress and use made of the project. We were concerned that projects studied would be described in the report and were therefore identifiable. As members of the advisory committee were prominent in the research community and had potential roles in future funding allocation we felt an obligation to protect respondents. As a result the advisory committee did not have access to tape recordings or transcripts, and interviewees had an opportunity to comment on what was said about their research in the draft report, before it was seen by the advisory committee.

### Analysis

The bibliometric properties of the journal articles derived from the projects were collated. Interviews were audio-taped and transcribed for thematic analysis using the Payback Framework and the Logic Model categories and scope notes [6]. Two grids were constructed. The first identified the impacts of each project in a range of categories, the second identified processes at each stage of the research which potentially led to outcomes. The NVivo 7 qualitative data analysis program was used to organise and analyse the data and the project team met regularly during the analysis of the data to facilitate agreement on the interpretation of the results.

## Results and discussion

This study was designed to trial the methodology as the first phase of a larger program of work. Accordingly, we report on the effectiveness of the data collection methods, the applicability of the Payback Framework, and on some issues which arose around the assessment of impact of primary health care research. To some extent we have framed our discussion in the context of a research impact framework by Kuruvilla et al which was developed in dialogue with the Payback Framework and was published after our project [12,13].

### Feasibility and effectiveness of data collection methods

The thirteen interviews conducted with researchers and nominated users provided more information about impact of these projects than the bibliometric analysis or documentary analysis. However, the tasks of scheduling, conducting and analysing the interviews were resource intensive. Although these projects were completed only two to six years before the interview, locating interviewees required some persistence including internet searches (5), repeated emails (39), and phone calls (19). One chief investigator responded only when contacted by the senior member of the research team. Five people had moved to new positions (one overseas) illustrating the workforce mobility of primary health care researchers in Australia. The average time taken from first contact until interview was 13 days, with a maximum of 26 days, as interviews had to be scheduled amongst the interviewees' other priorities, including extended leave. Arranging interviews with research team members was straightforward once the chief investigator had provided accurate contact details.

The response from users of the research was variable. The chief investigators contacted the users they nominated prior to providing the project team with names (but not in all cases contact details). Of ten potential users contacted by chief investigators, six were interviewed and one gave a brief statement. Those who took part had strong relationships with the chief investigators, which may have influenced their decisions, but the project team was not informed why the remaining three nominated users declined to be interviewed.

Interviews lasted from 60 to 90 minutes as interviewees, particularly researchers, were eager to talk about the impact of their work. Transcription took about 3 hours per hour of audiotape. The research team used NVivo to organise the material more systematically and compare the four projects. The initial process of coding and analysis in NVivo and compiling the case studies took approximately 15 days for the research officer who was familiar with the software, although this was an iterative process with writing and analysis undertaken in tandem.

The interviews became rather repetitive, partly because some interviewees addressed the content of later questions early on and partly because of overlap between some categories. For example, interviewees perceived overlap between two sub categories *Development of research skills, personnel and research capacity *and *Staff development and educational benefits*. The question "Was there an effect on the research team's capacity to use appropriate existing research from elsewhere?" was poorly understood and did not provide useful data.

The project documentation, media articles and research reports which CIs provided were more useful as evidence of dissemination than of research use or impact. Verifiable evidence of impact in the form of organisational documentation or policy documents was in most cases not available. This contrasts with Kuruvilla's study [13] which was able to locate confirmatory data in policy papers. Even records of dissemination other than reports and journal articles were patchy. Although all chief investigators reported in interviews they had presented the results of their work many times at conferences, seminars, meetings, and professional settings, only one was able to provide a list of conference presentations. Wooding et al [14] made similar observations on the lack of comprehensive records of presentations. Interpersonal connections, networks, committee participation and chance meetings which were important for dissemination and impact were hard to capture without adequate records.

The accurate list of journal publications required for bibliometric analysis was obtained more reliably from the chief investigator than from database searching, as it was difficult to identify papers which derive from a research project. However subsequent bibliometric analysis, which can provide evidence of use by researchers through citation numbers, was limited by the fact that only one of the seven peer reviewed papers resulting from these projects had been published in a journal indexed by the ISI Web of Science database, and with a recorded Impact Factor. Four of the seven articles were indexed by Scopus. This is consistent with the finding that analysis which depends on journal impact factors and citations may underestimate the payback for applied projects, in that no account is taken of journals not listed by ISI but which may be widely read by potential users such as clinicians [14,7]. Bibliometric analysis is known to be inconclusive and should be used with caution in primary health care research and in public health [9], which are published in a very wide range of journals [15] with patchy coverage in the ISI.

In summary, interviews provided valuable information about impact, but were time consuming to organise, conduct and analyse. More information was obtained from interviews with the chief investigators than from other team members. Interviews with research assistants and documentary sources provided little additional information. Interviews with users of research yielded rich information, however not all who were approached agreed to be interviewed.

Assessing impact of a substantial number of projects would be more feasible if the burden of response could be reduced by refining and streamlining the methods. Some possibilities include reducing the number of questions and overlap between them, reducing the amount of researcher time through use of a questionnaire, possibly web-based, followed if necessary by a brief phone interview, and surveying only one member of a research team [16].

### The Payback Framework

The four projects in this study were clinical or health services research projects, not basic science. Characteristics of each project and the availability of information in each of the Payback Framework categories for each project are shown in Table 1.

During the interviews, chief investigators provided more information about impacts in the first three payback categories (knowledge production; research targeting, capacity building and absorption; and informing practice and policy at a local or regional level) than about system wide, population or economic impacts (see Table 1). This is consistent with findings in the ARC study [6].

The interviews identified that the process of conducting these projects had strengthened relationships between the research team and their community and enhanced channels for future research impact. This could be regarded as an additional area of impact, not specifically included in the Payback Framework. The study by Kuruvilla et al [13] also found that all projects involved the formation and management of research collaborations and networks.

Interview questions relating to activities at the interface between research and potential end users at different stages of the research project (the Logic Model) revealed rich information about pathways to impact. This is summarised in Table 2.

In summary, the Payback Framework was applicable and very useful for structuring the data collection for primary health care research projects, despite overlap between two sub categories and the non-applicability of another. The scope could be enhanced to include strengthened connections for future research transfer to suit the collaborative nature of most primary health care research.

All these diverse projects fitted the payback categories, but some fitted the logic model of interfaces between the research team and the potential users better than others. The highly structured randomised controlled trial (Project 3) fitted the linear primary and secondary outputs and final outcomes more easily than the action research project (Project 1) in which practitioners and health service organisations informed and participated in the research at many stages and early findings were implemented during the project.

In completing the Payback Framework the research team encountered some dilemmas which are relevant to impact assessment generally.

### The extent of the respondents' knowledge of impact

As interviews turned out to the main source of information on impact in this study, much depended on researchers' extent of awareness of the impact of their project. This was greater in project 1 where the researcher remained in their position and was able to maintain contact with people who used the research, than in project 3 where both chief investigators had moved to different localities and the focus of their research interest had changed. Analysis suggested that chief investigators' awareness of impact, and possibly also the extent of assessed impact, may have been greater if they had documented interactions with potential users during their projects and followed up those which could potentially lead to impact. However, even if researchers maintain contact with potential users of their research and currency in their field of interest, they may be unaware of health and health sector benefits and economic benefits which are beyond their sphere of influence and awareness. Given the complexity of the interactions and chains of causalities in the process by which research influences policy and practice, the full extent of impact is unlikely to be revealed by interviews alone. Interviews with researchers and others were intended to be one of three interdependent methods of data collection used to build up a case study of impact [6] that would be supplemented by bibliometric analysis and documentary evidence of impact such as citations of research work in policy documents

### Evidence admissible in research impact assessment

It is unclear whether potential impact is admissible as evidence of impact. Respondents could describe plausible ways in which their findings could potentially lead to health and health sector benefits or broader economic benefit, but were not able to give evidence of actual benefits.

### Attribution

The unit of enquiry in this study was the funded research project, but respondents found it difficult to separate the effects of one project from those of the larger program of work in which they were involved. This is a feature of impact assessment rather than of the model itself. The impacts from several of the initial research projects were not separable from the impacts which occurred as a result of related organisational, policy or program development. It was not clear how many generations of impacts should be attributable to the research project.

A related issue was the difficulty of attributing long term impact to a single research project. Tracking research findings that are used instrumentally is more probable than those used for enlightenment or symbolically [17], because of the difficulty inherent in tracking changes in ideas and attitudes.

### Level of accountability

It is appropriate to study research impact using a project as the unit of enquiry only if it is valid to assume a single research study can or should prompt a change to health care or the health system. However, evidence based practice and evidence based policy making aim to base decision making on an accumulation of synthesised research evidence. Single research studies do not usually provide the standard of evidence required to make a change to professional practice, health care or the health system [18,19]. This was recognised in the Australian Research Quality Framework from 2005–2008 [20] which aimed to assess the impact of a body of work rather than of single projects. Attempting to assess the long term impact of individual projects in order to demonstrate accountability to a research funding body is therefore less meaningful than examining the impact of a body of work.

### Interpreting research impact

The impact of primary health care research projects needs to be assessed on a case by case basis, in relation to project intention and findings. The intervention arm of the randomised controlled trial (Project 3) in this study did not result in better outcomes than the control, so uptake of results was not to be expected. Project 2 which assessed quality of procedural care in rural practice did not find lack of quality, and therefore no major practice changes were required.

If research impact assessment shows little impact on policy development or the health sector, it would be incorrect to conclude that research was necessarily of poor quality or the funding was unjustified. Interviewees may have been unaware of impact, documentary evidence may have been unobtainable, or there may not have been time for the results to lead to impact. Uptake is a social and political process [4,21] influenced by many factors other than quality of research. The involvement of persons of influence or policy makers in the research process favoured uptake in projects 1 and 4. In project 3, the political context was initially receptive when the project was funded but had changed in response to other events leaving a research finding incongruent with other major influences and without impact, despite the best efforts of the research team.

## Conclusion

Interviews with chief investigators and users of research yielded rich and useful information on research impact, although they were resource intensive to organise, conduct and analyse. Bibliometric analysis was of little relevance in this sample due to only one journal being indexed in the ISI and having an impact factor. Documentary analysis provided evidence of dissemination rather than of impact.

The methods could be adapted for the next phase stage of this study by using survey methodology rather than interviews, and surveying only chief investigators. This has been found, by Buxton and Hanney [7] to provide a useful and reliable way to obtain an overview of the impact of funded research. A larger sample could provide useful ideas about activities and structures that encourage research use, as well as more substantial information on the categories of impact of primary health care research.

The categories in the Payback Framework were found to be applicable to assessing impact of primary health care research, especially the more proximal impacts on knowledge production, research targeting, capacity building and absorption, and informing practice, policy and product development. Much less information was available about the longer term categories of impact on health and health sector benefits and economics. The findings suggested an additional subcategory of impact, of strengthened networks for future research transfer. Although the Logic Model of interfaces between the research team and potential users was more suitable to a structured trial than to an action research project, it also provided the basis for useful information about how impact came about.

This trial has highlighted a number of issues with the assessment of research impact. The perspective of research team members and nominated users of research is limited to their own sphere of influence and awareness. Thought needs to be given to ways to differentiate impact resulting from research as opposed to policy and organisational development. Impact needs to be assessed in relation to project intentions, the nature of the research findings and the political climate.

## Competing interests

The authors declare that they have no competing interests.

## Authors' contributions

EK: assisted with the interviews, analysis and interpretation of the data and contributed to the manuscript. EJB conducted the interviews and bibliometric analysis, analysis and interpretation of the data and contributed to the manuscript. EM: assisted with the interviews, analysis and interpretation of the data and contributed to the manuscript. RR: analysed and interpreted the data and contributed to the manuscript. All authors read and approved the final manuscript.

## Appendix 1

### Schedule for semi structured interview with Chief Investigators

Could you tell me briefly about the aims of your project and what the findings were?

1. Do you know whether the findings have been used in any way?

2. Could you tell me about the peer reviewed publications which have derived from this project and where they have been published?

3. Are you aware of any papers that gave a citation to the papers you have published from your research? If yes, please give the reference if possible.

4. Could you tell me about any other publications such as reports to funders, or articles in press,

5. Have the results been featured in the media?

6. Have the results been disseminated elsewhere: specific conferences or seminars?

7. Are there any other outputs from this project?

### Organisational and research capacity

1. Has your research project led to any PhDs or other higher degrees for those working on the project, or is it likely to do so?

2. Has your project contributed to an increase in overall research capacity of your administrative unit?

3. Has this research project had any effect on your research team's capacity to use appropriate existing research from elsewhere?

4. Have the project findings or methodology generated subsequent research by members of the team?

5. Has your research had benefits in attracting further research funding?

6. Have the project findings or methodology generated or influenced subsequent research by other groups?

7. Has this project had any other staff development or educational benefits?

### Political and administrative impact

1. Has this project led to improved information by which to influence policy and executive decisions? Can you give examples?

2. Do you know if policy makers have used the results of this research project in any way? If yes, please give details.

3. Do you know if it has led to changes in policy?

4. Do you know if it has led to the development or refinement of any Government programs and initiatives?

5. Has it contributed/led to changes in knowledge, understanding and attitudes by policy makers? Has it been used to support arguments in a persuasive way?

6. Have there been other policy outcomes from this research?

### Health and health sector impact

1. Are you aware if your research has made any contribution to medical or allied health training?.

2. Are you aware of any impact that your research has had on health sector policy or practices, either directly, or through further research by your self or others?

3. Has this research led to any cost reduction in the delivery of existing services? Is there potential for this to happen in future?

4. Has it led (or might it lead) to any improvements in the process of service delivery?

5. Has it increased the quality or effectiveness of services?

6. Has this project led (or might it lead) to any other organisational development?

7. Has it led to changes in clinical practice by health practitioners?

8. Other health sector benefits?

### Consumer Outcomes

1. Has the research contributed to better health outcomes or improved quality of life for consumers at an individual or population level?

2. Has it had an effect (or might it) on equity?

3. Has it led to improved allocation of resources at an area level, better targeting or accessibility?

### Economic Outcomes

1. Have any revenues been gained from intellectual property rights?

2. Are you aware of any patents, or other commercial products, to which your research has contributed? If yes, please give details.

3. Is there any possibility of wider economic benefits from commercial exploitation of innovations from this research?

4. Has this research led to economic benefits from a healthy workforce and reduction in working days lost?

### Other outcomes

1. Have there been any other social, cultural or environmental outcomes from your project that we have not covered?

### Interface with potential users

2. Have the researchers worked with policy makers, practitioners or other potential users of research in any way before the project?

3. Have the researchers worked with key user groups during or after the project

4. Has your interface with potential users of the research impacted on how the results of your project have been used?

### Process issues

1. What organisational factors have influenced the dissemination and impact of your project?

2. What personal factors have influenced the publication and impact of the project?

3. Were any of the dissemination strategies particularly influential in achieving utilisation of the research findings? Why?

4. Please describe any other factors which have affected the impact of your research and any other outcomes not already covered.

### Potential or actual users of the research

1. In order to assess in more depth how your project has had an impact we would like you to suggest up to three people we could talk with who could provide a perspective on how the research has been used in policy, practice, organisational development, further research or in other applications such as guidelines or teaching materials. The research team will contact these people and interview them according to the developed protocol.

### Documentary sources

1. Would you be willing to provide a copy of the original project research proposal and any other project documentation which gives the original research question and/or the aims and objectives of the research?

We will be searching for documentary sources which could potentially provide evidence of impact. Are there any sources that you could tell me about?

## References

[B1] Australian Government Department of Health and Ageing (2005). Primary Health Care Research, Evaluation and Development Strategy Phase 2 (2006–2009) Strategic Plan.

[B2] Beacham B, Kalucy L, McIntyre E (2005). Focus on understanding and measuring research impact.

[B3] Davies H, Nutley S, Walter I (2005). Assessing the impact of social science research: conceptual, methodological and practical issues A background discussion paper for ESRC Symposium on Assessing Non-Academic Impact of Research May 2005.

[B4] Gabbay J, Le May A, Jefferson H, Webb D, Lovelock R, Powell J, Lathlean J (2003). A case study of knowledge management in multiagency consumer informed 'communities of practice': Implications for evidence based policy development in health and social services. Health.

[B5] Wells R, Whitworth J (2007). Assessing outcomes of health and medical research: do we measure what counts or count what we can measure?. Australia and New Zealand Health Policy.

[B6] Hanney S, Grant J, Wooding S, Buxton M (2004). Proposed methods for reviewing the outcomes of health research: the impact of funding by the UK's Arthritis Research Campaign. Health Research Policy and Systems.

[B7] Buxton M, Hanney S, Packwood T, Roberts S, Youll P (2000). Assessing the benefits from Department of Health and National Health Service Research and Development. Public Money and Management.

[B8] Kwan P, Johnston J, Fung A, Chong D, Collins R, Lo S (2007). A systematic evaluation of publicly funded health and health services research in Hong Kong. BMC Health Services Research.

[B9] Butler L, Biglia B (2001). Analysing the journal output of NH&MRC research grants.

[B10] Scopus database. http://www.elsevier.com/wps/find/bibliographicdatabasedescription.cws_home/705152/description#description.

[B11] Web of Science database. http://www.thomsonreuters.com/products_services/scientific/Web_of_Science.

[B12] Kuruvilla S, Mays N, Pleasand A, Walt G (2006). Describing the impact of health research: A research Impact framework. BMC Health Services Research.

[B13] Kuruvilla S, Mays N, Wait G (2007). Describing the impact of health services and policy research. Journal Health Services Research and Policy.

[B14] Wooding S, Hanney S, Buxton M, Grant J (2005). Payback arising from research funding: evaluation of the arthritis research campaign. Rheumatology.

[B15] Lowcay B, McIntyre E, Hale M, Ward A (2004). Peer reviewed publication rates: An indication of research output. Australian Family Physician.

[B16] Buxton M, Hanney S, Packwood T, Roberts S, Youll P (1999). Assessing the benefits from North Thames Research and Development. HERG Research Report No 25 Health Economics Research Group Brunel University, UK.

[B17] Amara N, Ouimet M, Landry R (2004). New Evidence on Instrumental, Conceptual, and Symbolic Utilization of University Research in Government Agencies. Science Communication.

[B18] Hanney S (2004). Personal interaction with researchers or detached synthesis of the evidence: Modelling the health policy paradox. Evaluation and Research in Evaluation.

[B19] Pakenham-Walsh N (2007). Wherever possible, research communication should be based on the scientific cumulation of knowledge, Response to Jonathan Lomas: The in-between world of knowledge brokering. BMJ.

[B20] Australian Government Department of Education Science and Training Research Quality Framework: Assessing the quality and impact of research in Australia: The recommended RQF. Australia.

[B21] Greenhalgh T, Russell J (2006). Reframing evidence synthesis as rhetorical action in the policy making drama. Healthc Policy.

